# Identification and Characterization of an O-Succinyl-L-Homoserine Sulfhydrylase From *Thioalkalivibrio sulfidiphilus*

**DOI:** 10.3389/fchem.2021.672414

**Published:** 2021-04-14

**Authors:** Wen-Yuan Zhu, Kun Niu, Peng Liu, Yu-Hang Fan, Zhi-Qiang Liu, Yu-Guo Zheng

**Affiliations:** ^1^The National and Local Joint Engineering Research Center for Biomanufacturing of Chiral Chemicals, Zhejiang University of Technology, Hangzhou, China; ^2^Key Laboratory of Bioorganic Synthesis of Zhejiang, College of Biotechnology and Bioengineering, Zhejiang University of Technology, Hangzhou, China

**Keywords:** O-succinyl-L-homoserine sulfhydrylase, cysthionine γ-synthase, L-methionine, biosyntheis, characterization

## Abstract

L-methionine is an important natural amino acid with broad application prospects. A novel gene encoding the enzyme with the ability to catalyze O-succinyl-L-homoserine (OSH) to L-methionine was screened and characterized. The recombinant O-succinyl-L-homoserine sulfhydrylase from *Thioalkalivibrio sulfidiphilus* (*ts*OSHS) exhibited maximum activity at 35°C and pH 6.5. OSHS displayed an excellent thermostability with a half-life of 21.72 h at 30°C. Furthermore, the activity of OSHS increased 115% after Fe^2+^ added. L-methionine was obtained with a total yield reaching 42.63 g/L under the concentration of O-succinyl-L-homoserine 400 mM (87.6 g/L). These results indicated that OSHS is a potential candidate for applying in the large-scale bioproduction of L-methionine.

## Highlights

- An enzyme with high activity that can convert O-succinyl-L-homoserine to L-methionine was screened.
- The protein sequence of the enzyme was identified.
- The enzymatic properties of the enzyme were studied and it has high thermostability.
- O-succinyl-L-homoserine sulfhydrylase was used in the L-methionine catalysis with high substrate concentration.

## Introduction

L-amino acid is the smallest component of protein, which is the basic life functional unit. L-amino acid has many important functions, such as providing nutrition for organism, protecting organism through immune system, participating in physiological metabolism process. In addition, with the understanding of L-amino acids, they are more and more used in food, pharmaceutical, cosmetics, feed additives and many other fields (Krämer, 2004). Among them, L-methionine is an important sulfur-containing essential amino acid, which participates in protein synthesis (Zhu et al., 2020). L-methionine is also the main methyl donor in many physiological metabolism processes, and the first limiting amino acid in animal nutrition (Tang et al., 2020). Much attentions have been paid to L-methionine as its tremendous demand used in various fields (Willke, 2014).

At present, L-methionine is mainly produced by chemical method. Methylthiopropionaldehyde was firstly synthesized from methyl mercaptan and acrolein, then hydantoin was synthesized from methylthiopropionaldehyde, HCN, NH_3_ and CO_2_. Hydantoin was then hydrolyzed to potassium methionine in the presence of KHCO_3_. Finally, methionine was obtained by acidification (Franke et al., 2003). In 1850, A. Strecker proposed that cyanohydrin was synthesized from acrolein, methyl mercaptan and hydrocyanic acid, and then hydrolyzed to hydroxymethionine and ammonium sulfate in the presence of sulfuric acid (Schonberg and Moubacher, 1952). In addition, the biological production of L-methionine was studied. Huang et al. rationally modified the metabolic pathway of *E. coli* W3110 by metabolic engineering technology to realize the production of L-methionine with a yield of 9.75 g/L (Huang et al., 2017). It has also been reported that *E. coli* MG1655 was developed to produce 35 g/L of L-methionine (Brazeau et al., 2013).

Besides, enzyme catalysis is also an important production route to obtain high yield of L-methionine. L-N-carbamoyl hydrolase was used to catalyze the cleavage of amide bond of N-carbamoyl methionine to produce L-methionine with the highest yield of 34.4% (Yamada et al., 1979). N-acetylamino acid racemase and amidase were coupled for racemization and transformation simultaneously to produce L-methionine with D, L-N-acetylmethionine as the substrate, the yield could reach 99% (Tokuyama and Hatano, 1996).

CJ Cheiljedang Co., Ltd. developed a route coupling fermentation and enzymatic catalysis, which used sucrose as a substrate to produce O-succinyl-L-homoserine (Hong et al., 2014) and O-acetyl-L-homoserine through fermentation. Then, it reacted with methyl mercaptan under the action of sulfhydrylase to produce L-methionine (Kim et al., 2015). At present, this route has the highest L-methionine production capacity through biological methods, and has already been industrialized.

Pyridoxal-5′-Phosphate (PLP)-dependent enzymes can catalyze various reactions in amino acid metabolism, including transamination, racemization, decarboxylation, and side chain elimination and replacement. At present, there are many enzymes belonging to the PLP-dependent enzyme superfamily (Christen and Mehta, 2001; Eliot and Kirsch, 2003). Among them, cystathionine synthase plays an important role in the sulfur metabolism pathways in plants, animals, and microorganisms (Zuhra et al., 2020). Cysthionine synthase could be divided into cysthionine β-synthase (α, β-elimination) and cysthionine-γ-synthase(α, γ-elimination) due to their different catalytic sites. Although both can catalyze the biosynthesis of L-cysthionine, they belong to the different folding types of PLP-dependent enzymes (Aitken and Kirsch, 2005). Cysthionine-β-Synthase (CBS) is the first and rate-limiting enzyme in the transsulfurization reaction and paticipated in many enzymatic processes (Mudd et al., 1965; Braunstein et al., 1969; Tudball and Reed, 1975; Kraus and Rosenberg, 1983). One of the features that distinguishes CBS from the other PLP-dependent enzymes is its N-terminus containing a heme-binding site (Zuhra et al., 2020).

Cysthionine-γ-Synthase (CGS) only existed in plants and bacteria (Aitken and Kirsch, 2005). It is a particularly interesting member of the γ-subfamily of fold-type I as it catalyzes a γ-replacement reaction that is unique among PLP-dependent enzymes (Brzovic et al., 1990). It is exhibited as the first specific enzyme for the methionine biosynthetic pathway. Nowadays, CGS has been extensively purified and characterized in *Salmonella thyphimurium, Escherichia coli*, and *Bacillus Sphcericus* (Kaplan and Flavin, 1966; Holbrook et al., 1990). However, the characterization of enzymatic properties directly applied to the synthesis of L-methionine *in vitro* has not been reported yet.

Therefore, in this study, 12 sequences with 50–90% homology were synthesized and overexpressed in *E. coli* BL21 (DE3). The sequence with the highest conversion rate was screened, and the enzymatic properties of OSHS were studied for the first time. OSHS with high thermostability, high activity, and high substrate tolerance and other characteristics, was exhibited the potentiality to be a biocatalyst for industrial production of L-methionine.

## Materials and Methods

### Chemicals

O-succinyl-L-homoserine and sodium methyl mercaptan were purchased from J&K Chemical Technology (Shanghai, China). L-methionine and PLP were purchased from Aladdin reagent Co. Ltd. (Shanghai, China). Kanamycin and isopropyl-β-D-thiogalactopyranoside (IPTG) were obtained from Sigma Chemical Co. (St. Louis, MO, USA). All other chemicals were of analytical grade and were commercially available.

### Plasmids, Bacterial Strains, and Culture Conditions

*E. coli* DH5α (TSINGKE Biotech Co., Hangzhou, China) was used as the host for gene cloning, and *E. coli* BL21(DE3) was the host for protein expression. The plasmid pET28b was used as the expression vector in *E. coli*. The *E. coli* was grown in Luria Bertani (LB) medium (yeast extract 5 g/L, peptone 10 g/L, NaCl 10 g/L) with 50 mg/L kanamycin.

### Screening, Identification, and Purification

Twelve genes were synthesized artificially according to the sequence deposited in the NCBI Protein database under accession number WP_016715902.1 (Kim et al., 2015). For expression purpose, the genes were subcloned to the expression vector pET28b between restriction sites NcoI and XhoI, and then the recombinant plasmids were transformed into the *E. coli* strain BL21(DE3). After the recombinant *E. coli* cells were cultivated in LB medium containing kanamycin (50 mg/L) at 37°C for 4 h, IPTG was added for induction and the cells were cultured at 28°C for 12 h. Recombinant OSHS was expressed intracellularly, and the pellet was collected by centrifugation at 12,000 rpm and 4°C for 10 min. To purify the recombinant OSHS, the centrifuged cell pellet was washed twice with 0.85% NaCl solution, and then resuspended in 50 mM sodium phosphate buffer (pH 7.0), and sonicated in ice bath. After sonication, the cell lysate was centrifuged at 8,000 rpm for 30 min at 4°C to remove cell debris. Then the supernatant was filtered through a 0.22 μm filter. The filtrate was further purified by a chelating agarose fast-flow resin column (1.0 × 10.0 cm) containing Ni2+, and the binding buffer (50 mM sodium phosphate buffer, pH 7.0) was used for balance. Then, proteins were eluted with a linear imidazole gradient of 20–500 mM in 50 mM sodium phosphate buffer (pH 7.0). The protein was analyzed by sodium dodecyl sulfate polyacrylamide gel electrophoresis (SDS-PAGE) to determine the protein purity. The protein concentration was determined by the protein determination kit (Keygen Biotech, China). Thereafter, the purified enzyme was used in characterization experiments. Its kinetics and catalytic properties were determined.

### Enzyme Assay

**Scheme 1:**
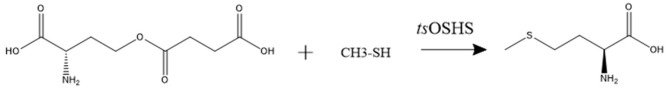
The reaction equation catalyzed by OSHS in this study.

The enzyme activity of recombinant OSHS was determined by measuring the conversion rate of OSH to L-methionine and the process was depicted by the reaction equation (Scheme 1). The system was carried out in a 500 μL system, which contained 50 mM Phosphate Buffered Saline (PBS) (pH 7.0), 50 mM OSH, 40 μL sodium methyl mercaptan and 15 μg purified enzyme at 30°C for 5 min. One unit (1U) is defined as the amount of enzyme catalyzing the production of 1 μM L-methionine from OSH per minute at 30°C. The L-methionine content was determined by amino acid analyzer.

### Effects of pH and Temperature on the Activity of OSHS

The influence of pH on the activity of the purified enzyme was performed at different pH (4.5–9.0). 100 mM OSH and 5% (v/v) sodium methyl mercaptan was biocatalyzed at 30°C for 10 min in three buffers with different pH range, citric acid-sodium citrate (pH 4.5–6.0), Na_2_HPO_4_-NaH_2_PO_4_ (pH 6.0-8), and Tris-HCl (pH 8.0–9.0). The pH stability was determined by measuring the residual activity after incubating the enzyme in buffers at pH 4.5–9.0 at 4°C for up to 10 h.

To investigate the optimal temperature, the reactions were carried out with 100 mM OSH, 5% (v/v) sodium methyl mercaptan, pH 7.0 adjusted with 50 mM PBS, and the temperature range was 20–60°C. The thermostability was analyzed by measuring the residual enzyme activity of the purified OSHS, which was incubated at 20 to 60°C for 25 h. The half-life of the enzyme was determined by calculating the change in Ln (relative activity) over time.

### Effects of Metal Ions and Organic Solvents on OSHS Activity

The effect of metal ions (CaCl_2_, MgSO_4_, FeCl_3_, FeCl_2_, CuSO_4_, MnCl_2_. ZnSO_4_, NiCl_2_, AlCl_3_, CoCl_2_) on purified OSHS activity were investigated at final concentrations of 1 and 5 mM, respectively. Effects of SDS, EDTA were investigated using the final concentration of 1 and 5 mM, respectively. All reactions were performed with 50 mM OSH, 10 mM PLP and 5% (v/v) sodium methyl mercaptan at 30°C, and the pH was adjusted to 6.5 with 50 mM PBS. The concentration of purified OSHS in the reaction was 30 mg/L.

### Kinetic Analysis

The kinetic parameters K_m_ (Michaelis-Menten constant) and V_max_ (maximum initial reaction rate) of OSHS were determined by using OSH as the substrate in the PBS at an initial rate ranging from 1 to 500 mM (pH 6.5). Before adding purified *ts*OSHS (30 mg/L), the reaction mixture was pre-incubated at 30°C for 10 min. The catalytic reaction was performed as a standard enzyme activity determination. K_m_ and k_cat_ were calculated by a non-linear regression method based on a function of the initial velocity data plotted against the substrate concentration.

### Biosynthesis of L-methionine Using Recombinant OSHS

Enzymatic synthesis of L-methionine was carried out in 200 of 500 mL shake flask under the optimum conditions. Samples were taken regularly to measure the concentration of L-methionine until the reaction reached equilibrium.

Purified OSHS enzyme with a final concentration of 30 mg/L was used to study the effect of substrate concentration on the enzymatic hydrolysis reaction in various concentrations of OSH (10–400 mM) in a reaction system with 10 mM PLP and 10% (v/v) sodium methyl mercaptan. The reactions were carried out at 30°C for 240 min. Under the condition of 400 mM OSH, 10 mM PLP and 10% (v/v) sodium methyl mercaptan, 30–100 mg/L purified OSHS was used to evaluate the effect of catalyst dosage on the enzymatic reaction. The reactions were carried out at 30°C for 240 min. Under the condition of 400 mM OSH, 100 mg/L purified OSHS and 10% (v/v) sodium methyl mercaptan, 1–20 mM PLP was used to investigate the effect of coenzyme on the catalyst. The reactions were carried out at 30°C for 140 min.

### Analytical Methods and Statistical Analysis

All the reactions were terminated using 100 μL 6 M HCl (10% v/v) for 10 min. The samples were diluted 100-fold and then centrifuged for 5 min at 12,000 rpm. Supernatant was collected and used for assay. Amino acids were detected using an automatic amino acid analyzer (SYKAM S-433D, SYKAM, München, BY, Germany) (El-Naggar et al., 2017; Huang et al., 2017). All experiments were replicated three times.

## Results and Discussion

### Gene Selection and Sequence Analysis

**Figure 1:**
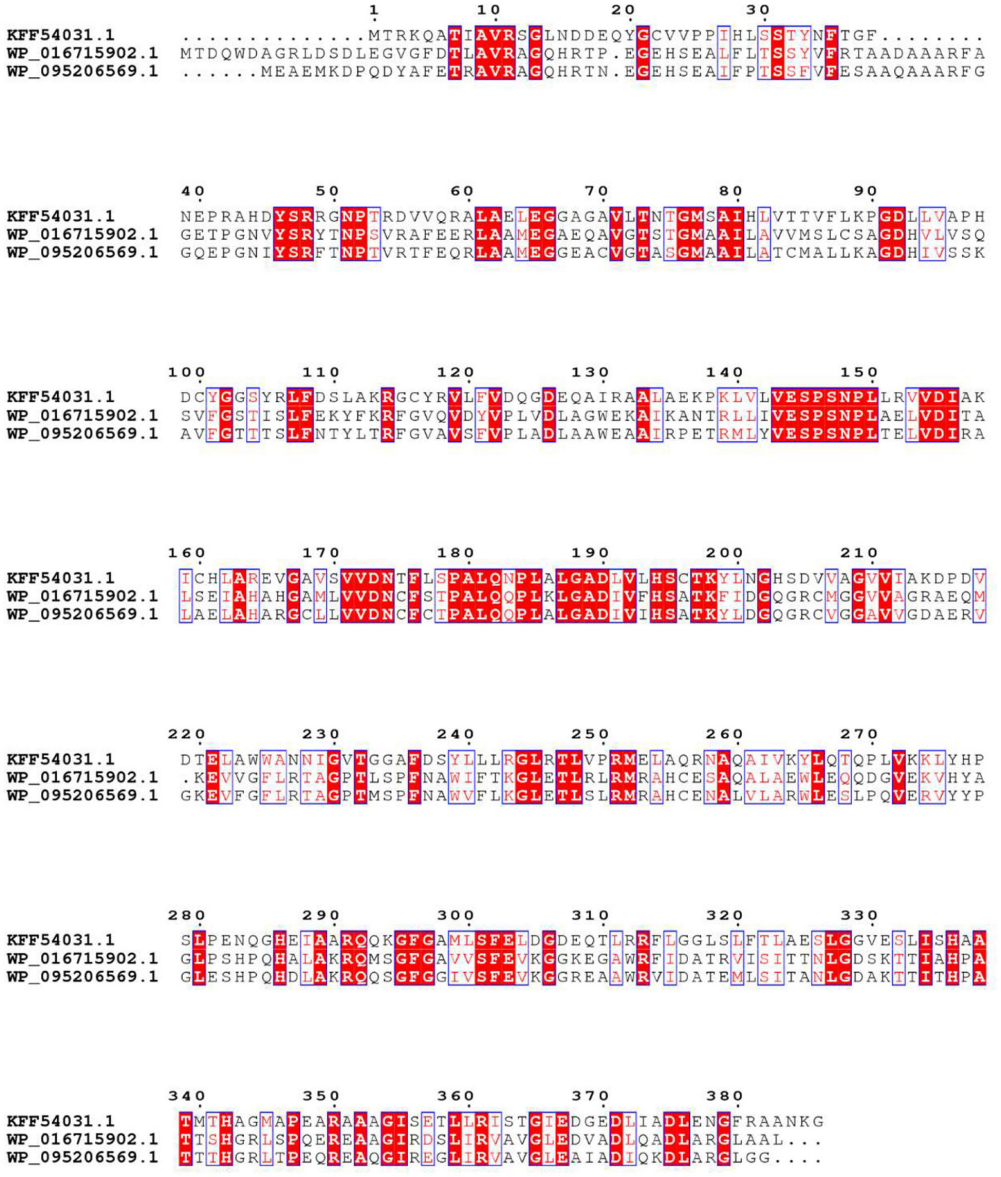
Alignment of the amino acid sequences. The origins and their GenBank NO.s are as follows: *E. coli* (KFF54031.1); *Pseudomonas* (WP_016715902.1); *T. sulfidiphilus* (WP_095206569.1).

In order to obtain an enzyme could convert OSH into L-methionine, gene mining approach was used to identify novel enzyme with high yield and the potential for industrial application. A reported OSHS from *Pseudomonas* could convert OSH into L-methionine (Kim et al., 2015) was used to perform a BLAST search in the nation center for biotechnology information database. The identity and conversion rate were listed in Table 1. Among them, WP_095206569.1 showed the highest conversion rate. Therefore, the protein sequence of this enzyme was aligned with the reported OSHS (WP_016715902.1) and one of the eCGS (KFF54031.1) (Figure 1). It was found that the three sequences shared the same conservative site in Y46, R48, R361, K198, and showed different in Y101 which was not directly involved in catalysis (Jaworski et al., 2012). Besides, the sequence analysis indicated that *ts*OSHS had a predicted molecular weight of 42736.1 Da and a predicted isoelectric point of 5.85.

**Table 1:** Identities and conversion rate of potential OSHS selected.

**Sequence ID**	**Identity**	**Conversion rate**
WP_043220698.1	79%	36.00%
WP_021218567.1	84%	41.51%
WP_095206569.1	71%	62.17%
WP_014852289.1	82%	49.29%
WP_061239023.1	82%	38.81%
PKM31047.1	75%	32.75%
WP_069562939.1	72%	45.31%
WP_038414333.1	81%	47.21%
EIJ33950.1	48%	5.08%
YP_009357725.1	46%	0%
AEG51937.1	54%	12.49%
AJY50471.1	45%	9.42%

### Phylogenetic Analysis of *ts*OSHS

**Figure 2:**
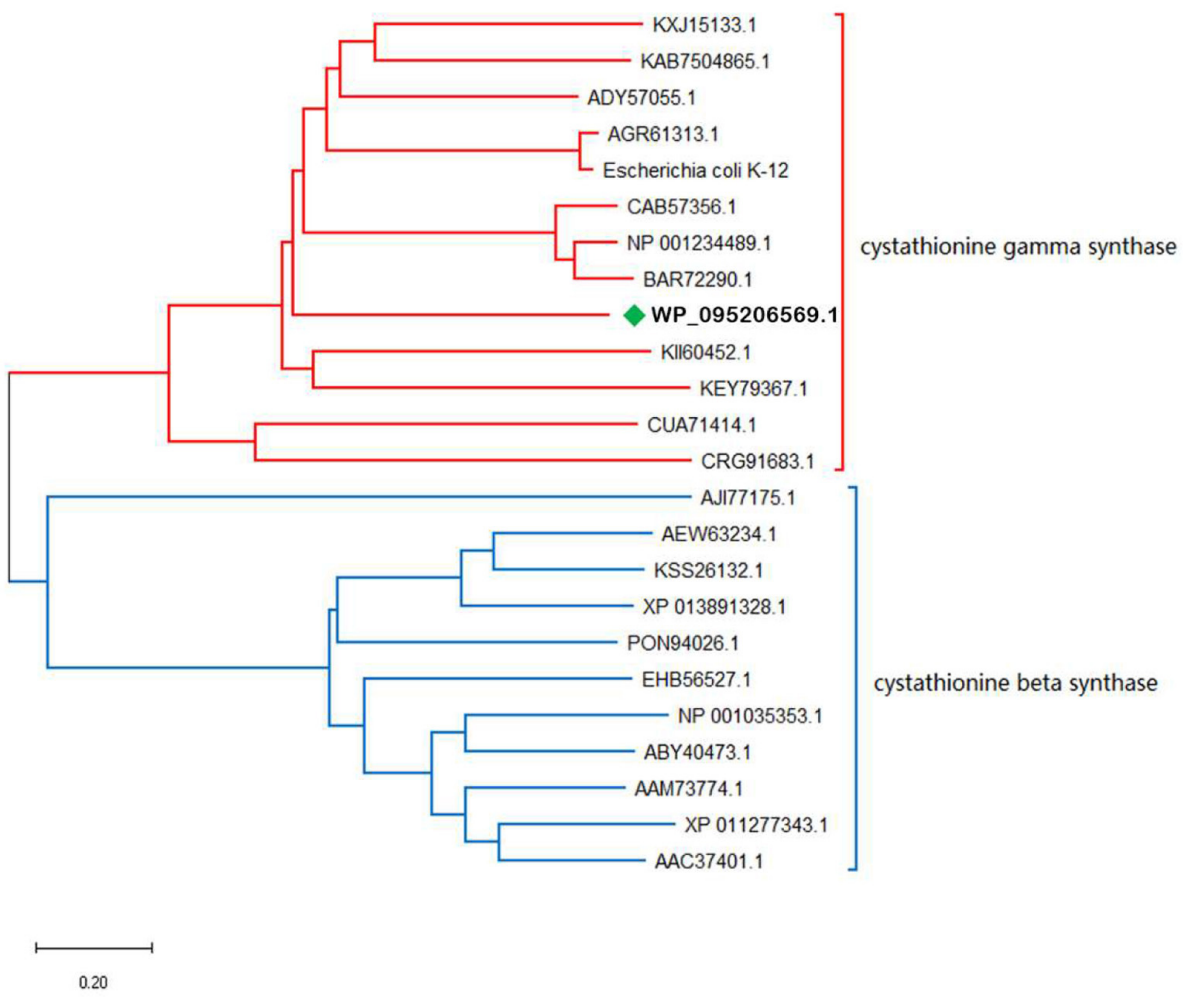
Phylogenetic analysis of OSHS and related enzymes.

OSHS showed 71% amino acid sequence identity with OSHS from *Thioalkalivibrio sulfidiphilus* (GenBank accession number WP_095206569.1). Blast analysis showed that *ts*OSHS belong to cystathionine synthase family and PLP-dependent enzyme superfamily which used PLP as the coenzyme for enzymatic reaction (Figure 2). The cystathionine synthase family can catalyze the α, β elimination and α, γ elimination. However, there is no corresponding report on the characteristics of OSHS in this reaction. Thirty sequences belonged to the cystathionine synthase were collected to analyze their phylogenetic relationship. The phylogenetic tree is divided into two obvious clusters, namely β elimination and γ elimination.

α, β-elimination mechanism is usually carried out through a quinone intermediate, in which the carbanion formed upon abstraction of the C_α_-proton is stabilized by delocalization into the pyridine ring of the PLP (Aitken and Kirsch, 2005). This transient intermediate can be detected when the elimination of the group is slow. All enzymes of the transsulfuration and reverse transsulfuration pathway except cysteine β-synthase are members of this subfamily. The structures of *E. coli* cysthionine-γ-Synthase (eCGS), yeast cystathionine c-lyase (yCGL) may originated from O-acetyl-L-homoserine sulfhydrylase, which were similar that their active sites located at the interface of subunits, and are composed of residues from two subunits (Messerschmidt et al., 2003). Phylogenetic analysis showed that *ts*OSHS is a member of the PLP-dependent enzyme superfamily cysteine γ-synthase.

### Gene Synthesis, Expression and Purification of OSHS

In order to functionally characterize the target enzyme, the synthesized *ts*OSHS gene was cloned into the pET28b expression vector. The 6 × His tag was added to the carbon end of the protein for affinity-based enzyme purification. The recombinant *ts*OSHS was purified from the supernatant of the cell lysate on a Ni-NTA column by one-step affinity chromatography. SDS-PAGE of the purified enzyme gave a single band showed a molecule of ~40 kDa (Supplementary Figure 1), which corresponded to the molecular weight calculated from the amino acid sequence obtained. The purified *ts*OSHS (318.2 U/mg) was then used for enzymatic characterization.

### Effects of pH and Temperature on *ts*OSHS Activities

**Figure 3:**
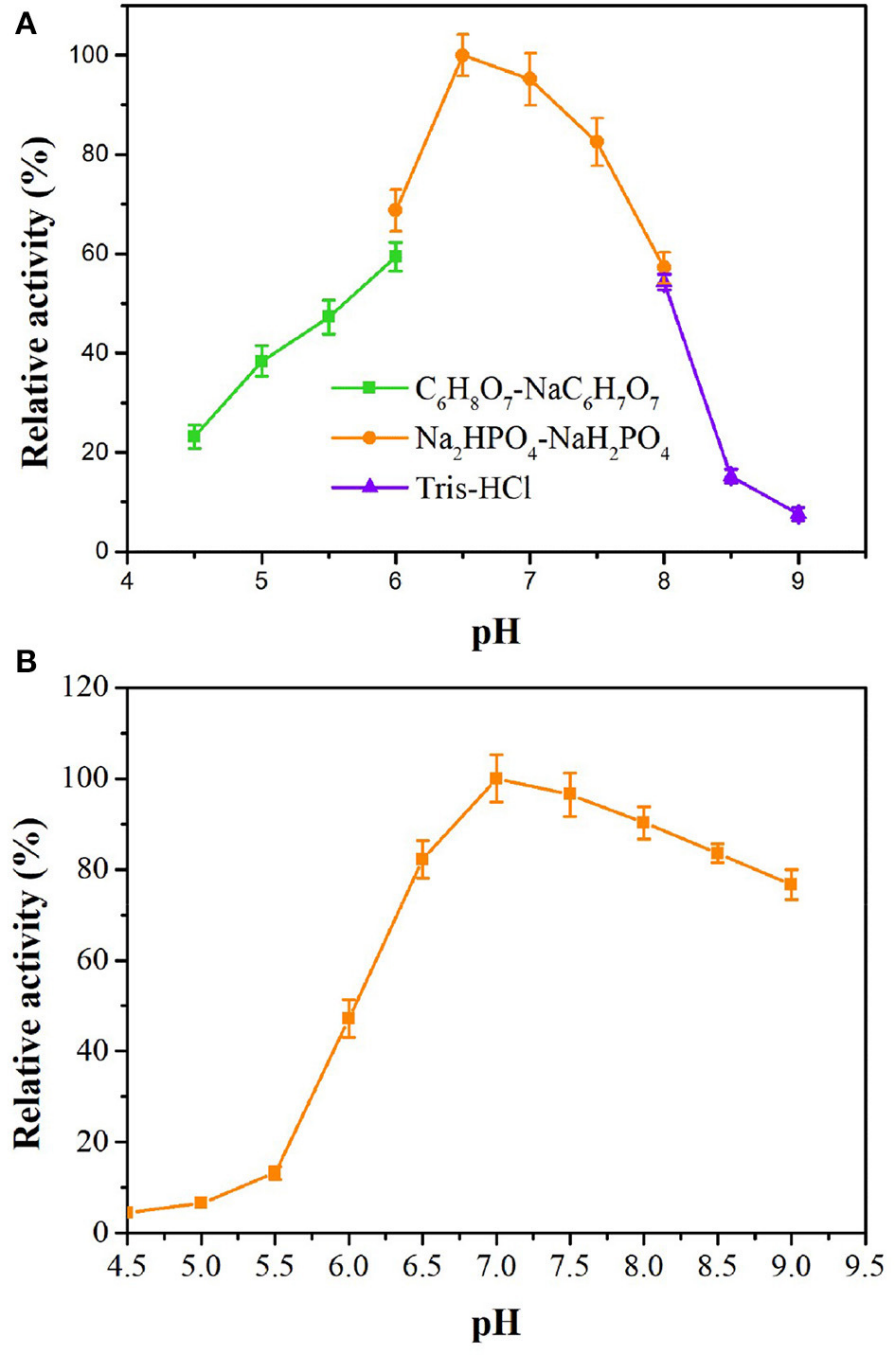
Effects of pH on enzyme activity of recombinant OSHS. The reactions were performed at 30°C for 10 min with 100 mM OSH and 5% (v/v) sodium methyl mercaptan. **(A)** pH optimization; **(B)** pH stability, the enzyme was incubated in buffers at pH 4.5–9.0 at 4°C for up to 10 h.

*ts*OSHS has high activity and stability in slight acid neutral buffer solution from pH 6.5 to 7.5, and the relative activity reached optimum at pH 6.5 (Figure 3), which indicated that *ts*OSHS has high activity in a certain pH range and has the potential of industrial application. However, the enzyme activity of *ts*OSHS decreased significantly when pH was higher than 7.5. In addition, the enzyme activity of *ts*OSHS in PBS was higher than that in other buffers. As shown in Figure 3, *ts*OSHS still maintained more than 80% activity in the pH range of 6.5–8.5 after 10 h incubation. The highest stability was achieved at pH 7.0 and the stability decreased significantly when pH was lower than 6.5, which indicated that the enzyme was unstable in acidic conditions. It could be found from the above results that the optimum pH and the pH maintain enzyme stability were more biased toward alkaline conditions. However, the enzyme activity reached the highest under neutral conditions. It could be explained that *ts*OSHS is derived from *T. sulfidiphilus* which is an alkaliphilic strain can maintain good stability in alkaline environment. In this study, *ts*OSHS showed better activity in neutral environment when it catalyzing OSH convert to L-methionine, as the substrate hydrolyzed spontaneously in alkaline environment which would cause that the detected enzyme activity lower than the actual enzyme activity.

**Figure 4:**
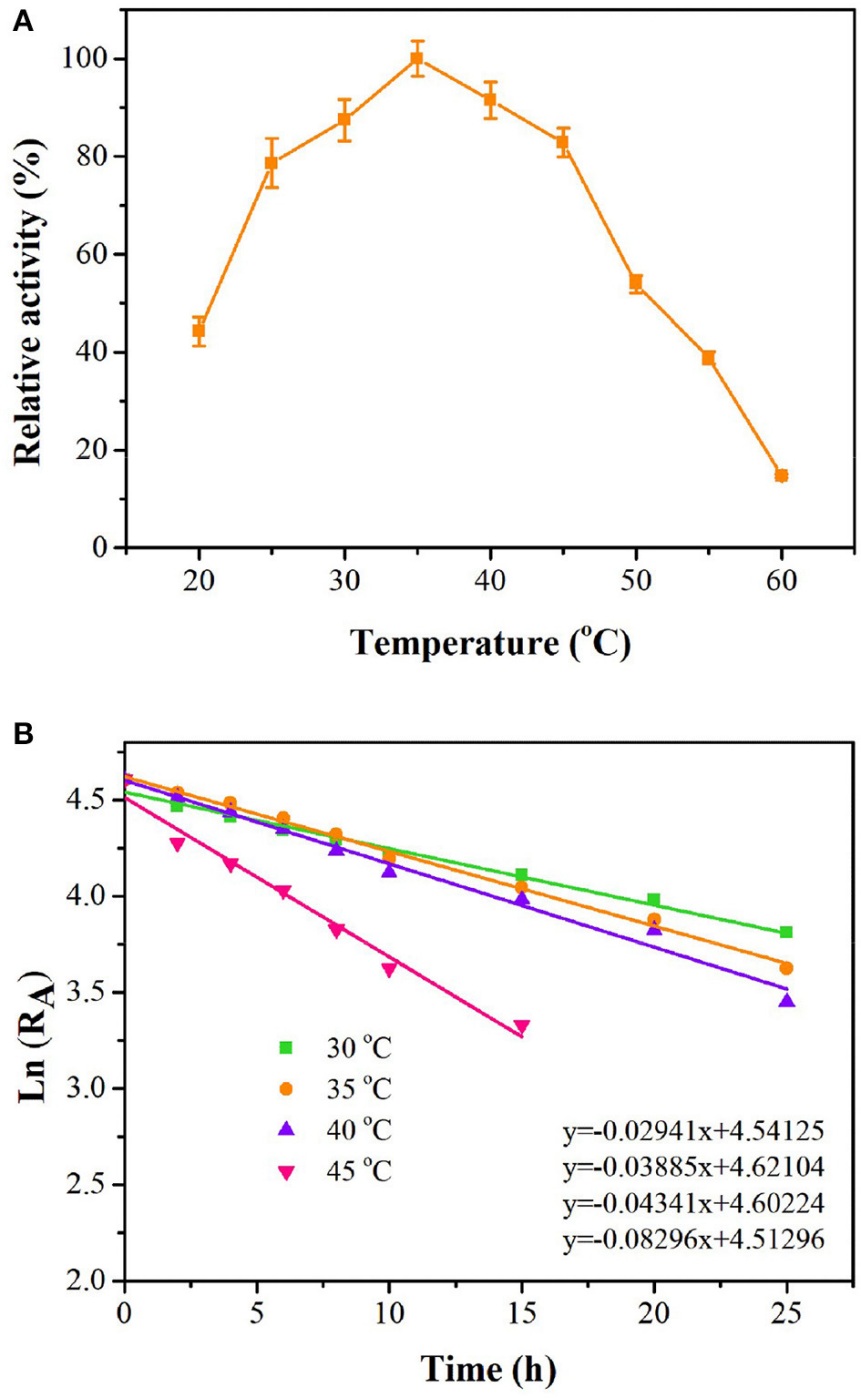
Effects of temperature on enzyme activity of recombinant OSHS. The reactions were performed at pH 7.0 for 10 min with 100 mM OSH and 5% (v/v) sodium methyl mercaptan. **(A)** Temperature optimization; **(B)** temperature stability, the enzyme was incubated in buffer at pH 7.0 at 20–60°C for up to 25 h.

As shown in Figure 4, *ts*OSHS had high enzyme activity at 25–45°C. The optimal temperature was 35°C, and there was still 91% of the optimal enzyme activity at 40°C. When the temperature increased over 45°C, the enzyme activity decreased rapidly, and almost no enzyme activity was detected at 60°C. The residual enzyme activity of *ts*OSHS was detected after incubation at 25–45°C in PBS (pH6.5). Accordingly, the half-life (t_1/2_) values were determined to be 21.72, 18.68, 16.05, and 7.32 h, respectively (Figure 4). These results indicated that *ts*OSHS had an excellent thermostability at 30–40°C, which make it a potential candidate for biocatalytic process development.

### Effects of Metal Ions and Organic Solvents

In order to explore whether *ts*OSHS has the properties of metalloenzymes, the effects of metal ions and organic solvents on enzyme activity were studied. The activity of purified *ts*OSHS affected by metal ions and organic solvents in the reaction were shown in Supplementary Figure 2. The activity of *ts*OSHS decreased with the increase of metal ion concentration. When the concentration of Cu^2+^ was 1 mM, the enzyme was seriously inhibited. When the concentration of Cu^2+^ and Al^3+^ was 5 mM, the enzyme was almost completely inhibited. Ni^2+^, Co^2+^, and Zn^2+^ also had a significant inhibitory effect on enzyme activity at 5 mM. In contrast, when the final concentration of Fe^2+^ was 1 mM, the activity increased 115%, but decreased when the concentration was 5 mM. And the effect of Fe^2+^ concentration on the relative activity of *ts*OSH was studied (Supplementary Figure 3). At the same time, the effects of 1 mM surfactant SDS and metal chelating agent EDTA on *ts*OSHS pre-treated with 1 mM Fe^2+^ were studied. The relative enzyme activity was 63 and 90%, respectively, which indicated that the enzyme activity showed inhibition under the reaction of organic solvents. Apparently, according to the above experiment results, various metal ions had almost no positive effect on *ts*OSHS, and the addition of metal chelating agents had no obvious inhibitory effect. Nowadays, there is no report showing that CGS has the characteristics of metalloenzymes. Therefore, it could be determined that *ts*OSHS is not a metalloenzyme.

### Kinetic Parameters

As shown in Supplementary Figure 4, the kinetic parameters were calculated through non-linear fitting results by plotting the initial velocity and OSH concentration. It was found that the V_max_, K_m_, and k_cat_ values of *ts*OSHS were 44.57 μmol min^−1^ mg^−1^, 72.52 mM and 29.71 s^−1^, respectively. It's higher than the K_m_ and k_cat_ of CGS from other sources producing cystathionine, such as 2.5 mM for *A. thaliana* CGS (Stephane et al., 1998), 3.02 mM for *H. pylori* CGS, and 1.29 mM for eCGS (Kong et al., 2008). It could be related to the structure of the sulfur donor. Compared with cysteine, methyl mercaptan used in this study leads to a smaller steric hindrance, so that the reaction of γ-elimination might be easier to happen.

### Biosynthesis of L-methionine Using Recombinant Purified *ts*OSHS

**Figure 5:**
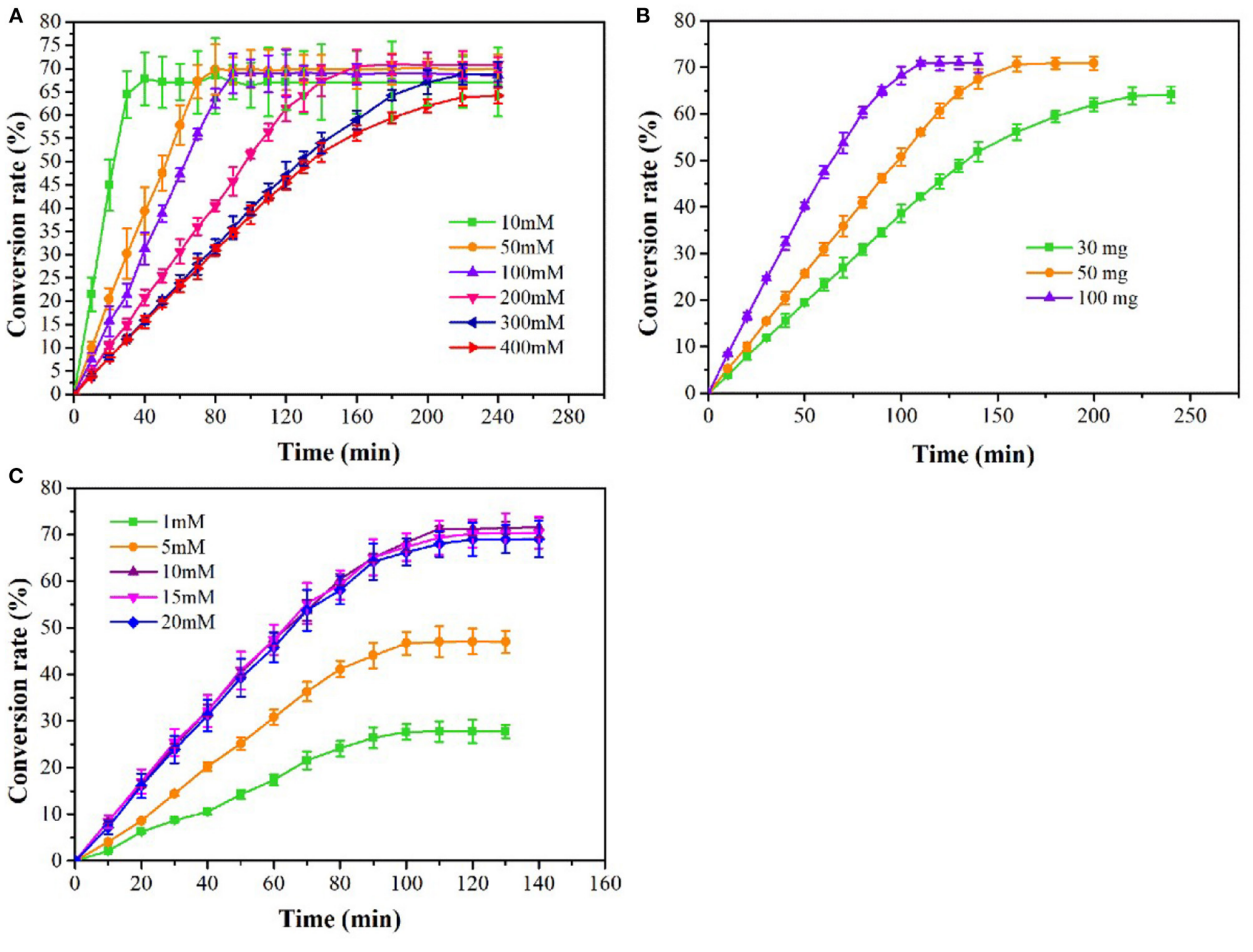
Effects on the production of L-methionine at different coditions. **(A)** The production of L-methionine at various substrate concentrations with 30 mg/L tsOSHS. The reactions were performed in the PBS (pH6.5) at 30°C with 10 mM PLP and 10% (v/v) sodium methyl mercaptan; **(B)** The production of L-methionine at various OSHS concentrations with 400 mM OSH. The reactions were performed in the PBS (pH 6.5) at 30°C with 10 mM PLP and 10% (v/v) sodium methyl mercaptan; **(C)** The production of L-methionine at various PLP concentrations with 400 mM OSH and 100 mg/L *ts*OSHS and 5% (v/v) sodium methyl mercaptan. The reactions were performed in the PBS (pH 6.5) at 30°C.

The effects of substrate concentration, enzyme concentration and PLP concentration on L-methionine production were studied. The yield of L-methionine was increased with the increasing of OSH concentration, and 38.25 g/L of L-methionine was obtained at 400 mM OSH reaching maximum yield (Figure 5A). With the extension of reaction time, the accumulation of L-methionine gradually slowed down and stabilized. When the substrate concentration was 400 mM, the conversion rate of OSH was 64.25% at 240 min and L-methionine was accumulated slowly. The results showed that the 30 mg/L *ts*OSHS could not completely catalyze 400 mM OSH. Therefore, in the following experiment, the concentration of *ts*OSHS in 400 mM OSH was optimized. As shown in Figure 5B, when the enzyme concentration increased from 30 to 100 mg/L, the accumulation rate of L-methionine increased. After about 120 min, 42.3 g/L-methionine was obtained, and the conversion rate increased from 64.17 to 71%. In addition, PLP is the coenzyme of *ts*OSHS and an important rate-limiting factor, which could affect the catalytic activity of *ts*OSHS. As shown in Figure 5C, when the concentration of PLP was lower than 10 mM, the accumulation amount and accumulation speed of L-methionine varied with the PLP concentration, indicating that the coenzyme concentration was not enough to maintain the catalytic activity of *ts*OSHS at this concentration. Besides, PLP not only has a catalytic role but also participate in the stability of CGS (Stephane et al., 1998). Therefore, when the PLP concentration increased, the conversion rate was significantly increased. When the concentration of PLP was over 10 mM, the yield of L-methionine and accumulation speed was close and the highest yield of L-methionine was 42.63 g/L. This indicated that when the concentration of PLP reached 10 mM, the ratio of enzyme and coenzyme was suitable for catalyzing the L-methionine production, and continued to increase the coenzyme concentration has almost no effect on the catalytic activity of the enzyme.

## Conclusions

In this study, a cystathionine synthase family *ts*OSHS with the activity to catalyze OSH to L-methionine screened from NCBI by blast was synthesized and expressed in *E. coli* BL21 (DE3). The recombinant enzyme was purified by one-step affinity chromatography on Ni-NTA column. The properties of purified *ts*OSHS were studied. The recombinant *ts*OSHS reached highest activity at pH 6.5 and 35°C. It exhibited excellent thermostability with a half-life of 21.72 h at 30°C. Trace Fe^2+^ could promote the activity of *ts*OSHS, but the promotion is limited. Under the optimal conditions, the substrate concentration, enzyme amount and coenzyme amount were optimized, and finally 42.63 g/L L-methionine was obtained, and the conversion rate was 71%.

In conclusion, this is the first report on the synthesis, expression and enzymatic properties of OSHS which used in the L-methionine production and the high substrate tolerance, high conversion rate, high productivity (511.56 g/L/d) and high yield of L-methionine demonstrated the high potential of *ts*OSHS in large-scale biosynthesis of L-methionine.

## Data Availability Statement

The original contributions presented in the study are included in the article/Supplementary Material, further inquiries can be directed to the corresponding author.

## Author Contributions

Z-QL and Y-GZ initiated and supervised the project. W-YZ carried out all data analyses. W-YZ and KN wrote and modified the manuscript. PL and Y-HF conceived the study, participated in designing, and coordinating the study. All authors read and approved the final manuscript.

## Conflict of Interest

The authors declare that the research was conducted in the absence of any commercial or financial relationships that could be construed as a potential conflict of interest.
