# Supplementary material for: Identification and Characterization of an O-Succinyl-L-Homoserine Sulfhydrylase From Thioalkalivibrio sulfidiphilus
Source: Front Chem. 2021 Apr 14;9:672414. doi: 10.3389/fchem.2021.672414 (PMC8080516; doi:10.3389/fchem.2021.672414)
Supplement: Supplementary file 1 [file Data_Sheet_1.docx]

**Figure S1** Verification of the expression and molecular weight of the OSHS sequence synthesized.  Lane M, protein marker; Lane *ts*OSHS, purified recombinant *ts*OSHS.

**Figure S2** Effects of metal ions and chemical solvents on recombinant *ts*OSHS. The reactions were performed at pH 6.5 and 30 ℃ for 30 min with 50 mM OSH, 10 mM PLP and 5 % (v/v) sodium methyl mercaptan.

**Figure S3** Effects of Fe^2+^ concentration on the relative activity of *ts*OSH. The reactions were performed at pH 6.5 and 30 ℃ for 30 min with 50 mM OSH, 10 mM PLP and 5 % (v/v) sodium methyl mercaptan. Fe^2+^ were added ranging from 0-5 mM.

**Figure S4** Nonlinear regression of Michaelis-Menten. The reactions were performed in the PBS (pH 6.5) with 10 mM PLP at an initial rate ranging from 1 to 500 mM (pH 6.5) with purified *ts*OSHS (30 mg/L) at 30 ℃ for 10 min.

Figure S1


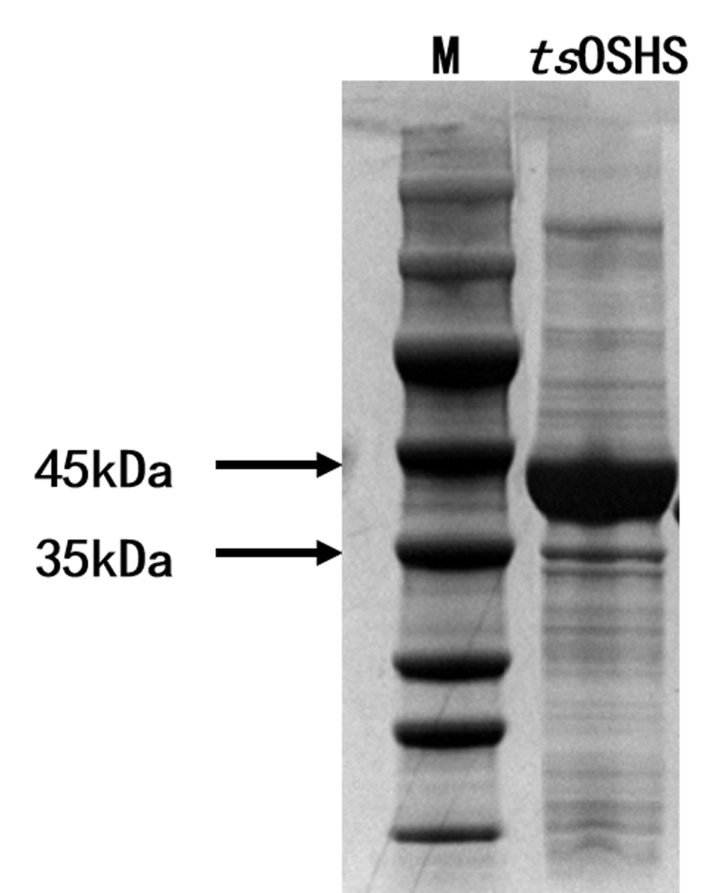


Figure S2





Figure S3





Figure S4
